# P-1651. Utilizing Electronic Medical Record (EMR) Based Tools to Shorten Duration of Therapy for Community Acquired Pneumonia in the Ambulatory Setting

**DOI:** 10.1093/ofid/ofae631.1817

**Published:** 2025-01-29

**Authors:** Shankar Upadhyayula, Kelly Williamson

**Affiliations:** Akron childrens hospital, PENINSULA, Ohio; Akron childrens hospital, PENINSULA, Ohio

## Abstract

**Background:**

Community acquired pneumonia (CAP) is a commonly encountered diagnosis is the pediatric outpatient setting. Short durations of antimicrobial therapy of 5-7 days are considered adequate for uncomplicated CAP. We wanted to utilize Electronic medical record (EMR) based tools to standardize treatment durations for CAP. Akron children’s hospital is a large pediatric health care provider in Northeast Ohio and offers pediatric care through more than 30 pediatric offices in the community. This extensive network of primary care offices affords unique opportunities to use the EMR based tools for care standardization.

Counts of antibiotic orders based on duration.

**Figure:**
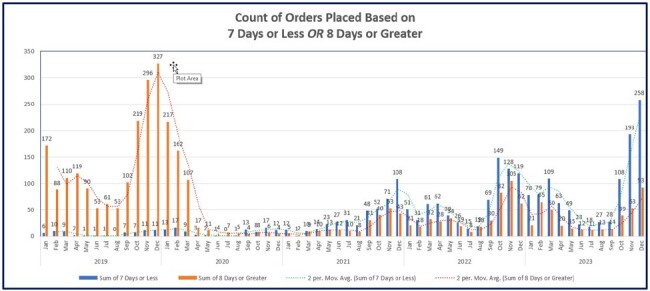

**Methods:**

The antimicrobial stewardship committee developed a guideline for diagnosing and managing community acquired pneumonia and distributed it to all primary care providers. A standardized smart set provided clinical decision support regarding choice of antibiotics and duration of therapy. A visit diagnosis of Community acquired pneumonia prompted the use of the smart set. Smart set usage and duration of therapy (7 days or less vs 8 days or more) were monitored. Baseline data for one year prior and 3 years post-introduction were tracked to measure impact and monitor sustainability of change.

Percentage of antibiotic orders based on duration.

**Figure:**
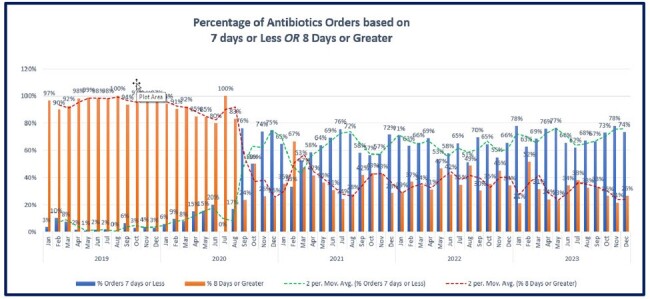

**Results:**

The smart set went live in January 2020. Data review from January 2019 to December 2023 showed the percentage of orders with antibiotic prescriptions of 8 days or more decreased from 97% to 34% while 7 days or less increased from 3% to 66% (Figures 1,2). This change is associated with a high utilization of the pneumonia smart set of > 70 percent (Figure 3). Even though the project is in its maintenance phase the gains have sustained over time.

Smart set usage for antibiotic orders

**Figure:**
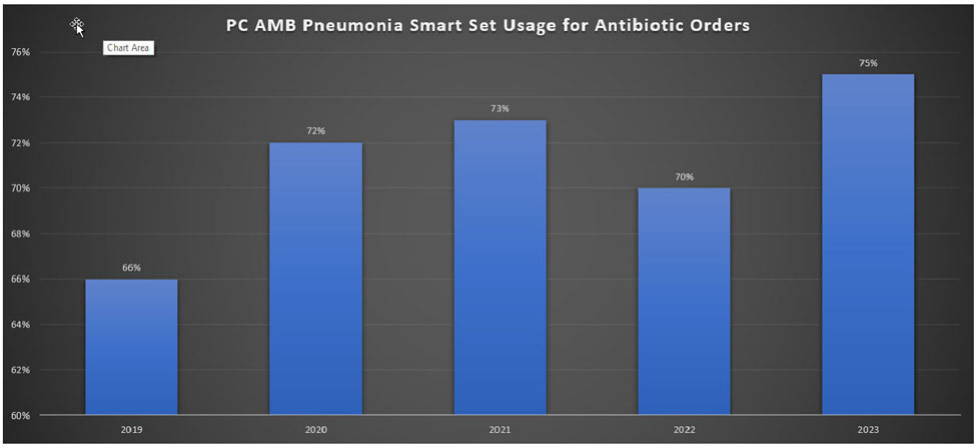

**Conclusion:**

Utilizing EMR based tools can help shorten antibiotic duration for community acquired pneumonia in a sustainable fashion. This approach holds promise as it can be easily utilized to standardize care for other common outpatient conditions. Expectedly the strategy would be most applicable to health care networks with a wide outreach. It also requires intensive education of providers at the outset, but this can be accomplished by using interested primary care providers to partner with antimicrobial stewardship teams.

**Disclosures:**

**All Authors**: No reported disclosures

